# Examining the effect of Medicaid expansion on early detection of head and neck cancer of the oral cavity and pharynx by HPV‐type and generosity of dental benefits

**DOI:** 10.1002/cnr2.1840

**Published:** 2023-05-29

**Authors:** Jason Semprini

**Affiliations:** ^1^ Department of Health Management and Policy University of Iowa College of Public Health Iowa City Iowa USA

**Keywords:** head and neck cancer, oral cancer, screening, SEER, statistical methods

## Abstract

**Background:**

Over a decade of evidence supports the claim that increased access to insurance through Medicaid expansions improves early detection of cancer. Yet, evidence linking Medicaid expansions to early detection of head and neck cancers (HNC) of the oral cavity and pharynx, specifically, may be limited by the lack of attention to Human Papillomavirus (HPV) etiology, generosity of dental coverage, and valid inference analyzing state cancer registry data.

**Aims:**

This study reexamined the effect of Medicaid expansion on early detection of HPV+/− HNC in states offering extensive dental benefits.

**Materials and Methods:**

Specialized data from the Surveillance, Epidemiology, and End Results (SEER) program was analyzed to account for, previously unmeasurable, differential detection patterns of HNCs associated with HPV. Then, to identify the effect of increasing Medicaid eligibility on staging patterns in states offering extensive benefits amidst potentially non‐common trends between states, a “Triple Differences” design identifies the differential effect of Medicaid Expansion (with dental coverage) on HPV‐negative HNCs relative to the change in HPV‐positive HNCs. For valid inference analyzing a small number of state clusters (12) in cancer registry data, each regression model applies a Wild Cluster Bootstrap.

**Results:**

Expanding Medicaid eligibility was found to be associated with a decrease in the proportion of distant‐stage diagnoses of HPV(−) HNCs, but only among states which increased Medicaid dental generosity at the time of Medicaid expansion.

**Conclusions:**

These results suggest that adding extensive Medicaid dental benefits was the primary mechanism impacting HNC detection. This study highlights the potential positive spillover effects of policies which increase access to public dental coverage for low‐income adults, while also showing the limitation of access to dental services for improving early detection of HPV+ HNCs.

## 1 INTRODUCTION

This paper reexamines the potential impact of new access to affordable Medicaid insurance coverage on staging outcomes for head and neck cancers of the oral cavity and pharynx (HNC). Specifically, this study investigates the Affordable Care Act's (ACA) Medicaid expansions, a policy which has been rigorously studied for nearly a decade.^1^ Studies have generally found that by increasing access to physicians, Medicaid expansions improved physician‐based cancer screenings and early‐stage cancer diagnoses of cancers with systematic screening protocols.^2^ However, the limited research on HNC has not resulted in clear consensus, or sufficiently explained the potential mechanisms linking access to Medicaid with improved staging outcomes.^3^, ^4^, ^5^, ^6^

Comprising over 90% of all head and neck cancers, HNCs of the oral cavity and pharynx kill more than 10 000 adults each year.^7^, ^8^, ^9^ Compared to localized‐stage HNCs, a distant‐stage diagnosis can lower the probability of 5‐year survival by 40%–70%.^9^ Late detection also raises healthcare expenditures and exacerbates financial toxicity. The annual treatment cost‐per‐person for late‐stage HNCs is $10 000 higher than the standard of care for early‐stage HNCs.^10^, ^11^ In 2019, less than 30% of HNCs were diagnosed at localized stages.^8^, ^9^ Policies that increase the proportion of HNCs detected early could dramatically improve outcomes for this rare, but deadly disease.

Two recent studies found mixed evidence that Medicaid Expansion improved HNC detection. The first study analyzed hospital‐based data to find that stage I‐II diagnoses of non‐oropharyngeal HNC increased in Medicaid Expansion states relative to non‐Expansion states.^12^ The second study analyzed population‐based data to find that Medicaid Expansion was associated with higher rates of early‐stage diagnoses, but only in low‐incidence populations (i.e., young adults, females).^13^ Both studies used traditional Difference‐in‐Differences designs, but neither study empirically examined their analytical models for bias. More critically, these prior studies relied on the fundamental assumption that, upon gaining access to Medicaid coverage, low‐income adults at risk of developing HNC were widely screened by physicians, largely ignoring the role of dentists in state Medicaid dental systems.

Dental services are a critical component of HNC detection. In the United States, dental professionals are among the only healthcare provider recommended to systematically conduct visual oral cancer examinations for adult patients.^14^, ^15^, ^16^, ^17^ This is likely why dentists conduct the overwhelming majority or HNC screenings.^18^ Unfortunately for efforts to improve population oral health, financial barriers prohibit adequate utilization of dental services.^19^, ^20^ Medicaid has served as a critical access point for low‐income adults, but barriers persist. Adult Medicaid dental benefits remain optional and, therefore highly volatile. The consequences of this variation in access to Medicaid dental services continues to be of great concern for policymakers and investigators.

If the ACA's Medicaid expansions were to improve HNC detection, the most likely pathway would be through increased access to Medicaid dental services and dentist‐based HNC screenings. However, HNCs comprise a heterogenous group of malignant tumors.^8^, ^21^ The efficacy of discriminatory screening varies not just by tumor site, but by Human Papillomavirus (HPV) etiology.^22^ HNCs caused by HPV, which now account for the bulk of all HNCs, are less likely to be detected early by visual examination.^8^, ^12^, ^23^ Researchers and clinicians continue to develop and test novel approaches for detecting HPV+ HNCs at earlier stages.^24^, ^25^, ^26^, ^27^ However, current HPV‐associated oropharyngeal cancer screening protocols are currently not justified in the population.^28^ HNCs not associated with HPV, rather, can be screened for and identified by a visual examination during a comprehensive oral exam; an exam which is most often performed by a dental professional.^18^, ^29^

### 1.1 Objective

This study tests the hypothesis that Medicaid Expansion with extensive dental benefits impacts early detection by increasing access to dentist‐based HNC screening, but only where screening is most valuable (HNCs not associated with HPV).

## 2 METHODOLOGY

### 2.1 Data and sample selection

This study is among the first to evaluate the ACA by analyzing the Specialized Head and Neck Cancer (HPV) data file from the Surveillance, Epidemiology, and End Results (SEER) program.^30^ The specialized data file includes a clinically validated variable indicating the HPV etiology (HPV in situ hybridization, tissue polymerase chain reaction, in situ hybridization for E6/7 RNA, real‐time polymerase chain reaction for E6/7 RNA) of each HNC tumor within the following HNC sites: Lip, Hypopharynx, Nasopharynx, Oropharynx, Pharyngeal Tonsil, Pharynx Other, Palate Soft, Tongue Base, Gum and Other Mouth, and Other Oral Cavity.^30^ Following established practice, missing HPV‐status was imputed with data on tumor grade, histology, site, behavior, and encrypted census tract‐level socioeconomic indicators via a logistic regression model through Multiple Imputation Chained Equations.^31^, ^32^, ^33^, ^34^, ^35^, ^36^, ^37^, ^38^ This specialized data includes cancer diagnoses for year 2010–2016. Because adults over age 65 could benefit from access to Medicaid dental coverage, the analytical sample includes adults between ages 30–84.

### 2.2 Design

#### 2.2.1 Treatment assignment

Medicaid expansion is the treatment of interest. Adults diagnosed with HNC in Medicaid expansion states are assigned into the treatment group (California, Washington, Iowa, Kentucky, New Jersey, Connecticut, Hawaii, New Mexico, and Michigan) and adults diagnosed with HNC in non‐expansion states are assigned into the control group (Georgia, Louisiana, and Utah). This unstaggered design is among the most common approaches used to study the effects of the ACA's Medicaid expansion and the approach used by the two recent studies on HNC detection.^12^, ^13^ Each treatment state offered extensive Medicaid dental benefits at the time of expansion in 2014.^39^ Although, California and Washington added extensive dental benefits in 2014.^40^, ^41^ All other states did not change their Medicaid dental benefits during this time.

The final strategy tests if adding Medicaid dental coverage or increasing Medicaid eligibility is the critical mechanism impacting HNC outcomes. Here, the two states which added extensive Medicaid dental benefits in 2014 are considered the treated group and all other states as controls. To estimate the association between adding dental benefits and HNC outcomes, this study tests for differential changes between groups before and after 2014. Then, to test if increasing Medicaid eligibility (without dental benefits) was associated with HNC outcomes, this study tests for differential changes before and after 2012 (when both CA and WA began implementing Medicaid expansion) for California and Washington compared to all other states; this test excludes the years 2014–2016.^42^

#### 2.2.2 Variables

The primary outcomes of interest are derived from the SEER Combined Summary Stage variable, which categorizes the presentation of each tumor as localized, regional, and distant.^43^ The first outcome is a binary variable indicating if the HNC patient was diagnosed at a localized stage. The second outcome is a binary variable indicating if the HNC patient was diagnosed at a distant stage. Medicaid coverage serves as a secondary outcome.^44^ To account for temporal trends and unobserved heterogeneity, all models control for year and state fixed‐effects. The model also accounts for seasonal variation by including month dummy variables. All models control for patient age, race/ethnicity, sex, marital status, tumor site, and metro status.

#### 2.2.3 Empirical strategy

As a linear probability regression model, a Difference‐in‐Differences design identifies the association between Medicaid expansion and HNC outcomes. The initial specification aims, in part, to replicate prior evidence of Medicaid expansion's association with the probability of an early‐stage HNC diagnosis. Stratification analyses test for differences by HPV status and the timing of extensive Medicaid dental coverage. The final specification constructs a Triple Differences Model, which identifies the association between Medicaid expansion with dental benefits and HNC outcomes for HPV(−) HNCs, relative to the association between Medicaid expansion and HNC outcomes for HPV+ HNCs. This third difference accounts for non‐common trends between expansion and non‐expansion states, as long as those non‐common trends are consistent between HPV+ and HPV(−) HNCs.

For all models, an event‐history study tests for pre‐expansion differential trends in HNC outcomes between expanding and non‐expanding states. See Appendix A1 for more details on these identification assumption and pre‐trend tests.

### 2.3 Statistical analysis and inference

Given that the treatment is assigned at the state‐level, theory and common practice suggest that inference should utilize standard errors clustered at the state‐level to account for within‐state correlation. However, because of the limited number of states participating in SEER, there are insufficient clusters for valid, robust inference. To mediate this threat to inference, this study implements robust Wild Bootstrapping procedures following each model.^45^ Using Webb resampling weights to further ensure robustness against type 1 error due to small number of clusters, 999 replicate bootstraps with standard errors clustered at the state‐level construct a 95% confidence interval set.^45^ Statistical significance is set at alpha = .05. All analyses were conducted using STATA v. 17.

## 3 RESULTS

### 3.1 Summary statistics

**FIGURE 1:**
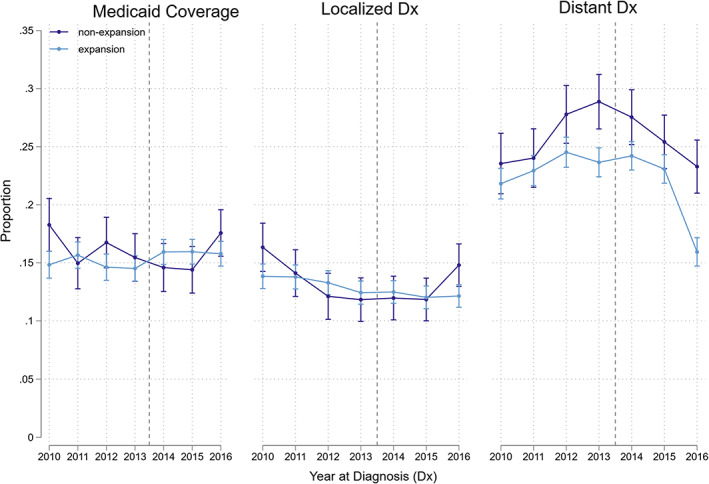
Medicaid and staging trends by expansion status. This figure visually depicts the year‐by‐year proportion of all HNC patients by the proportion covered by Medicaid (left), diagnosed early (middle), and diagnosed late (right). The vertical dotted lines represent the respective expansion years for early and late expanding states. *Y*‐axis reports proportions on a 0–1 scale. Dx, diagnosis.

**FIGURE 2:**
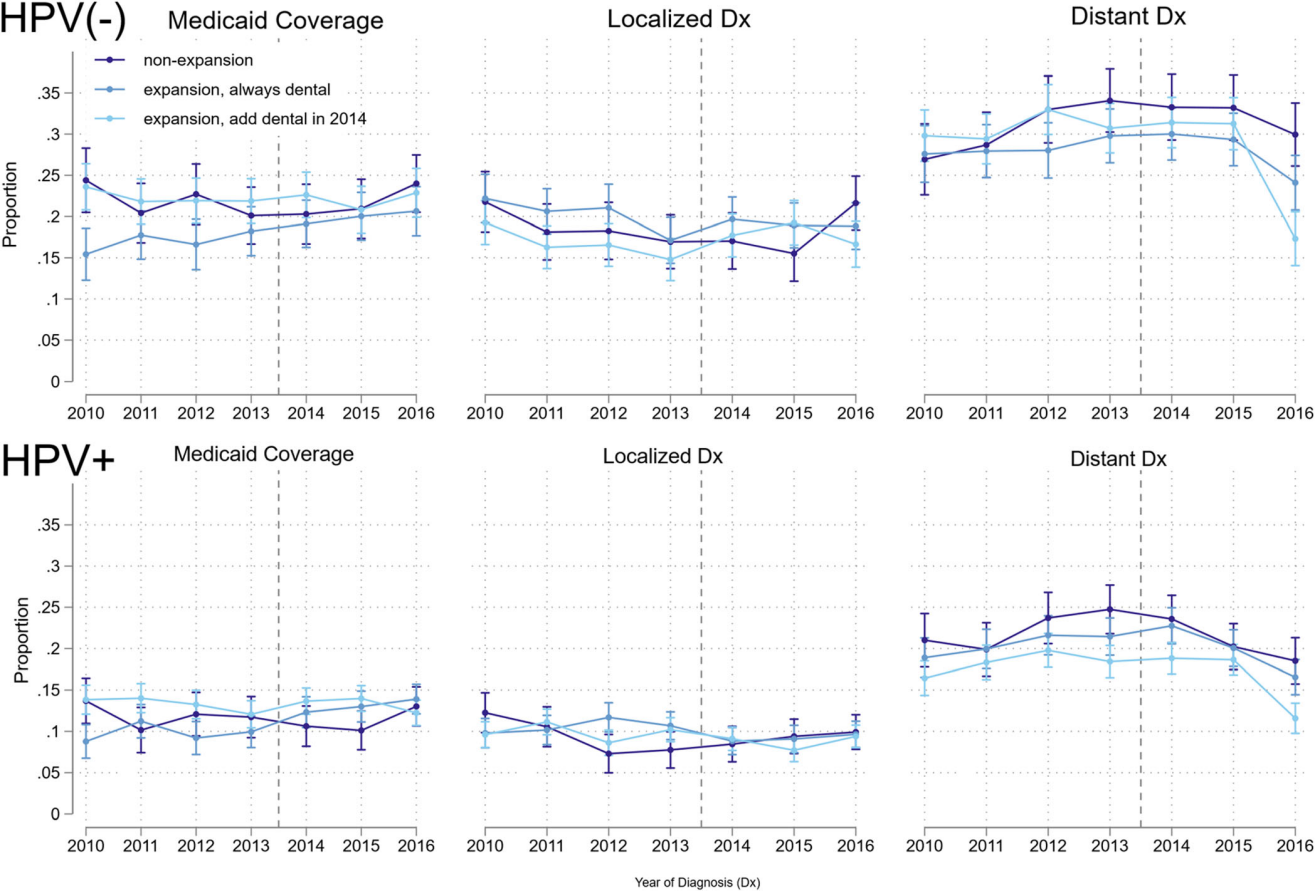
Medicaid and staging trends by expansion, HPV, and dental status. This figure visually depicts the year‐by‐year proportion of all HNC patients by the proportion covered by Medicaid (left), diagnosed early (middle), and diagnosed late (right). This figure shows the trends stratified by HPV(−) (top) and HPV+ (bottom), and timing of dental coverage: “always” versus “adding dental in 2014” (California, Washington). The vertical dotted lines represent the respective expansion years for early and late expanding states. *Y*‐axis reports proportions on a 0–1 scale. Dx, diagnosis.

The analytical sample contains 38 123 adults diagnosed with HNC. In the pre‐expansion period (2010–2013), only 12.4%–14.4% of the sample's HNCs were diagnosed at localized stages. Figure 1 shows the unadjusted, annual HNC outcome trends by expansion status. Figure 2 further stratifies the sample by HPV‐status and timing of state Medicaid dental generosity. See Table 1 for sample descriptive statistics.

**TABLE 1:** Descriptive statistics of head and neck cancer patients (SEER HNC‐HPV).

	Non‐expansion	Expansion, Group 1	Expansion, Group 2
Medicaid coverage	0.163	0.127	0.152
Localized Dx	0.135	0.144	0.124
Distant Dx	0.262	0.236	0.229
HPV(−) status	0.445	0.400	0.374
Age	60.0	60.6	60.9
Male	0.797	0.804	0.810
Non‐Hispanic White	0.751	0.810	0.710
Married	0.529	0.534	0.540
Tongue site	0.291	0.299	0.317
Tonsil site	0.354	0.358	0.355
Metro	0.801	0.816	0.951
*N*‐sample	2970	4763	5874
*m*‐states	3	9	2

*Note*: This table reports the 2010–2013 summary statistics (means) for three groups. Groups were assigned by state‐level Medicaid expansion and dental coverage decisions. Expansion, Group 1 are states which expanded Medicaid in 2014 and always covered extensive Medicaid dental services throughout the study period. Expansion, Group 2 (California, Washington) are states which expanded Medicaid in 2014 but did not cover extensive Medicaid dental services until 2014. The *N*‐sample reports the total number of HNC patients in each of the groups for all years 2010–2016. The average age is reported in years. All other averages are reported on a 0–1 scale. For proportions, the denominator are all HNC patients within each respective group (column).

Abbreviation: Dx, diagnosis.

### 3.2 Empirical results

In the full sample, Medicaid expansion was associated with a 2.46 percentage point increase in the probability of Medicaid coverage (CI = [−0.0164, 0.1018]; *p* = .0821), but had a near‐zero and statistically insignificant association with localized stage diagnoses (Table 1: Est. = −0.0074; CI = [−0.0221, 0.0112] and distant stage diagnoses; Est. = −0.0055; CI = [−0.094, 0.0326]).

#### 3.2.1 Expansion in states always covering dental benefits

Regarding Medicaid coverage take up in states always covering generous Medicaid dental benefits, this study estimates statistically significant and clinically meaningful associations between expansion and Medicaid coverage. The 3.85‐percentage point estimate (CI = [0.0045, 0.0808]; *p* = .044) represents a 30% relative increase in Medicaid coverage from the baseline mean. The 3.97‐percentage point estimate for adults with HPV+ HNC represents a 40% relative increase from baseline, but the joint pre‐trend tests suggest this result be interpreted with caution (Supplemental Table 1; *p* = .0360). Yet, despite the estimated increase in Medicaid coverage for HNC patients in states always covering extensive dental benefits, there is no association between Medicaid expansion and staging at diagnosis. This finding is supported by the Triple Differences estimates and by the insignificant pre‐trend test statistics (Supplemental Tables 2–6).

#### 3.2.2 Expansion in states adding dental coverage in 2014

Similar to the estimates in the states always covering generous Medicaid dental benefits, in states adding generous dental coverage at the time of expansion there also appears to be a significant and positive association between expansion and Medicaid coverage (Est. = 0.0139; CI = [0.0059, 0.0478]; *p* = .026). However, in the states adding dental benefits there is a statistically significant change in Medicaid coverage for adults with HPV+ HNC. The 1.88‐percentage point estimate (CI = [0.004, 0.0697]; *p* = .012) in HPV+ adults represents a 14% relative increase in Medicaid coverage rates. The estimate for HPV(−) HNC patients is half the size in magnitude (Est. = 0.0079) and not statistically different than zero (CI = [−0.0513, 0.0621]).

Contrary to the results in states always covering dental benefits, and despite no evidence of Medicaid take up after expansion, this study does identify a marginally significant association between Medicaid expansion and distant‐stage diagnoses in states adding dental coverage in 2014. Here, expansion was associated with a 4.24‐percentage point decline in distant‐stage diagnoses (CI = −0.2602, 0.0036; *p* = .059), a 19% relative decline from pre‐expansion rates. The Triple Differences estimate for HPV(−) HNCs is similar (Table 3: Est. = 0.0460; CI = [−0.3067, 0.0026]; *p* = .0581).

Estimates for localized diagnoses for HPV+ HNCs in these states may be threatened by differential pre‐expansion trends (Supplemental Table 2). For this reason, the DDD estimates for HPV(−) may not be valid (Supplemental Table 5). However, there is no evidence from the DD or DDD pre‐trend tests to suggest differential trends in distant‐stage diagnoses for HPV(−) HNCs prior to expansion in these states (Supplemental Tables 3 and 6).

#### 3.2.3 Adding dental coverage in 2014

Adding extensive Medicaid dental benefits was not associated with changing Medicaid coverage (Tables 2 and 3). However, adding extensive Medicaid dental coverage was associated with lower rates of distant‐stage diagnoses in the full sample (Est. = −0.0151; CI = [−0.0374, 0.0016]; *p* = .0611). The estimate for HPV+ HNCs was near zero and statistically insignificant. However, the DD‐estimate for HPV(−) HNCs was statistically significant (Est. = −0.0337; CI = [−0.1181, −0.0053]; *p* = .0360). The estimates also reveal that adding extensive Medicaid dental benefits was associated with a 2.02‐percentage point increase in the probability of localized‐stage diagnoses among HPV(−) HNCs (CI = [0.0049, 0.0701]; *p* = .0350). This estimate corresponds to a 16.3% relative increase in the probability of localized‐stage diagnoses relative to 2013 rates. The DDD‐estimates yield similar results in terms of magnitude and inference, but the DDD estimate for localized diagnoses may be threatened by significant pre‐trends (Supplemental Table 8). The DD and DDD estimates for the distant‐stage diagnoses are supported by the lack of significant pre‐trend test statistics (Supplemental Tables 7 and 8). None of the estimates for the association between expanding Medicaid in 2012 (without dental benefits) and HNC staging were even marginally statistically significant.

**TABLE 2:** Medicaid expansion's association with HNC outcomes (DD).

		Medicaid coverage	Localized Dx	Distant Dx
		Est. [CI]	Est. [CI]	Est. [CI]
Expansion	All	0.0246*	−0.0074	−0.0055
		[−0.0164, 0.1018]	[−0.0221, 0.0112]	[−0.0904, 0.0326]
	HPV(−)	0.0193	0.0065	−0.0279
		[−0.0283, 0.1048]	[−0.0298, 0.0857]	[−0.1020, 0.0149]
	HPV+	0.0275**	−0.0152	0.0096
		[−0.0011, 0.1071]	[−0.0249, 0.0089]	[−0.0733, 0.0649]
Expansion, always dental coverage	All	0.0385**	−0.0119	0.0034
		[−0.0045, 0.0808]	[−0.0263, 0.0121]	[−0.0231, 0.0378]
	HPV(−)	0.0330*	−0.0055	−0.0123
		[−0.0210, 0.0880]	[−0.0364, 0.0333]	[−0.0585, 0.0468]
	HPV+	0.0397**	−0.0177	0.0121
		[0.0052, 0.0863]	[−0.0354, 0.0147]	[−0.0069, 0.0435]
Expansion, adding dental coverage in 2014	All	0.0139**	−0.0032	−0.0126
		[0.0059, 0.0478]	[−0.0055, 0.0049]	[−0.0313, 0.0272]
	HPV(−)	0.0079	0.0176	−0.0424*
		[−0.0513, 0.0621]	[−0.0118, 0.1686]	[−0.2602, 0.0036]
	HPV+	0.0188***	−0.0126	0.0068
		[0.0041, 0.0697]	[−0.0533, 0.0151]	[−0.0149, 0.0951]
Adding dental coverage in 2014	All	−0.0104	0.0041	−0.0151*
		[−0.0549, 0.0357]	[−0.0142, 0.0226]	[−0.0374, 0.0016]
	HPV(−)	−0.0121	0.0202**	−0.0337**
		[−0.0732, 0.0488]	[0.0049, 0.0701]	[−0.1281, −0.0053]
	HPV+	−0.0077	−0.0008	−0.0021
		[−0.0545, 0.0391]	[−0.0298, 0.0239]	[−0.0181, 0.0398]
Early expansion in 2012 (no dental coverage)	All	−0.005	−0.0015	−0.0053
		[−0.0481, 0.0454]	[−0.0231, 0.0393]	[−0.0291, 0.0253]
	HPV(−)	−0.0108	0.0001	−0.0045
		[−0.0989, 0.0791]	[−0.0314, 0.0476]	[−0.0504, 0.0880]
	HPV+	−0.0019	−0.0067	−0.0062
		[−0.0319, 0.0275]	[−0.0430, 0.0428]	[−0.0316, 0.0228]

*Note*: Est. = Difference‐in‐Differences (DD) estimates of expanding Medicaid eligibility in 2014. CI = 95% confidence interval from robust wild bootstrap procedures. Each two‐way fixed effect linear probability regression model was estimated separately and stratified by HPV‐status and timing of offering extensive Medicaid dental benefits. (1) Expansion compares expansion versus non‐expansion states; (2) expansion, always dental coverage compares expansion states (excluding California and Washington) with non‐expansion states; (3) expansion, adding dental coverage compares expansions states (only including California and Washington) with non‐expansion states; (4) adding dental coverage in 2014 compares California and Washington with all other states; (5) early expansion in 2012 serves as a placebo test to compare trends before and after 2012 in California and Washington with all other states.

Abbreviation: Dx, diagnosis.

* *p* < .1;

** *p* < .05;

*** *p* < 0.01. [Correction added on 27 June 2023, after first online publication: Table 2 and 3 footnote ‘***’ value has been corrected in this version.].

**TABLE 3:** Medicaid expansion's association with HPV(−) HNC outcomes (DDD).

	Medicaid coverage	Localized Dx	Distant Dx
	Est. [CI]	Est. [CI]	Est. [CI]
Expansion	−0.0108	0.0204	−0.0318*
	[−0.0395, 0.0425]	[−0.0273, 0.1531]	[−0.1284, 0.0015]
Expansion, always dental coverage	−0.0091	0.0124	−0.0215
	[−0.0429, 0.0489]	[−0.0373, 0.0665]	[−0.0615, 0.0149]
Expansion, adding dental coverage in 2014	−0.0117	0.0269	−0.0406*
	[−0.0749, 0.0579]	[−0.0214, 0.2391]	[−0.3067, 0.0026]
Adding dental coverage in 2014	−0.0061	0.0187*	−0.0262*
	[−0.0498, 0.0316]	[−0.0027, 0.0789]	[−0.1472, 0.0032]
Early expansion in 2012 (no dental coverage)	−0.0098	0.0091	0.0006
	[−0.0792, 0.0374]	[−0.0639, 0.0906]	[−0.0581, 0.0854]

*Note*: Est. = Triple Differences (DDD) estimates of expanding Medicaid eligibility for HPV(−) HNCs in 2014. CI = 95% confidence interval from robust wild bootstrap procedures. Each two‐way fixed effect linear probability regression model was estimated separately and stratified by HPV‐status and timing of offering extensive Medicaid dental benefits. (1) Expansion compares expansion versus non‐expansion states; (2) expansion, always dental coverage compares expansion states (excluding California and Washington) with non‐expansion states; (3) expansion, adding dental coverage compares expansions states (only including California and Washington) with non‐expansion states; (4) adding dental coverage in 2014 compares California and Washington with all other states; (5) early expansion in 2012 serves as a placebo test to compare trends before and after 2012 in California and Washington with all other states.

* *p* < .1; **p < .05; ***p < 0.01. [Correction added on 27 June 2023, after first online publication: Table 2 and 3 footnote ‘***’ value has been corrected in this version.].

Abbreviation: Dx, diagnosis.

## 4 DISCUSSION

Medicaid expansion under the Affordable Care Act may have reduced the proportion of HNCs diagnosed at late stages, but only for patients whose cancer was not caused by HPV and who lived in California and Washington state who increased their generosity of Medicaid dental benefits at the time of expansion. Conversely, there does not appear to be any signal of an association between Medicaid expansion and HNC detection in states which expanded Medicaid but always covered generous dental benefits. Neither does there appear to be any improvement in HNC staging for HPV+ HNCs, despite the strong evidence that patients with HPV+ HNC were more likely to be covered by Medicaid insurance as a result of expansion.

Placed in the context with the other studies on Medicaid expansion and HNC detection, these findings are, in some ways, similar. Like other studies, this study found no overall association between expanding Medicaid and staging outcomes at the population level. Similarly, this study only found an association between expanding Medicaid with specific subpopulations.^12^, ^13^ Perhaps the subgroups of these prior studies were, in fact, proxies for HPV(−) HNC. Note the Sineshaw study includes HPV‐stratification as a supplemental aim, and this study affirms their hospital‐based results at the population‐level.^12^ Viewed together, three studies now suggest that Medicaid expansion had some positive impact on earlier HNC detection, but only for certain adults. This work argues that those certain adults were patients with HPV(−) HNCs who gained access to extensive Medicaid dental services. Until future research extends the design using more years and more states, these results should only be generalized to California and Washington state adults, among which the vast majority of the sample were male, non‐Hispanic White, and metro county residents.

To further contextualize these results, it is important to assess the validity of this study's inference. Here, the discussion extends beyond studies investigating Medicaid expansion and HNC, but all quasi‐experimental, state‐based policy research analyzing cancer registry data. In most of the cancer research, including the HNC studies motivating this work, estimating valid standard errors receives little detail or consideration. More concerning was the extensive work evaluating Medicaid expansion on a wide array of cancers and outcomes using cancer registry data; data that contains between 12 and 22 clusters of states which vary considerably by both size and composition. Applied and theoretical econometricians have developed methodologies to obtain valid inference under such circumstances. A primary example is the Wild Bootstrap Clustering algorithm. Yet, few Medicaid expansion studies have implemented these procedures and even fewer appear to have implemented the Wild Bootstrap Clustering algorithm to infer an unbiased effect of Medicaid expansion analyzing SEER cancer registry data. The implications of this gap are currently unknown. Perhaps the evidence‐base would be unchanged with more attention to rigorous inference methodology. This discussion serves as a call to replicate and validate existing and future research. By improving rigor, trust, and transparency, our field can continue to improve our ability to inform policy decisions with observational research.

As of January 2023, all but 11 states have expanded Medicaid eligibility through the ACA.^46^ According to the latest MACPAC report, 14 states do not cover any preventative dental services to the adult population.^39^ Most states today operate their Medicaid dental program via a managed care system, each implementing different strategies to control oral health care and expenditures.^47^ There is a well‐established, causal relationship between access to generous Medicaid dental benefits and higher utilization of dental services.^48^, ^49^ Undoubtedly, by preventing low‐income adults from accessing affordable health insurance, the persistence of non‐expansion states likely contributes to health inequities.^50^ This current study further illuminates the potential inequities in HNC detection that result from heterogenous and volatile access to Medicaid dental services.

### 4.1 Limitations

This study is not without its limitations. First and foremost, all analyses are subject to limited Power to detect effects given that HNC is, by definition, a rare cancer. The lack of Power hinders any ability to further stratify the sample by site or other patient demographic features. Future work could expand upon this study to investigate how expanding access to dental services impacts oral health equity. Further, while the SEER cancer registries account for nearly half of all cancers diagnosed in America each year, the HNC‐HPV SEER datafile contains only 12 unique clusters of states. This is problematic for state‐based quasi‐experimental research, again from a Power and inference perspective, not to mention testing for differential pretreatment trends with fewer years of data. This study followed best practices for valid identification and inference to accommodate the limited sample and number of clusters.^45^, ^51^, ^52^, ^53^ Also related to data limitations are the potential for mismeasured HPV‐status in this novel specialized registry datafile.^30^ However, while the potential missingness may be correlated with time, there is no evidence that this potential missingness is correlated with Medicaid Expansion status over time. Additionally, these results were consistent with preliminary work which only analyzed cancer cases with known HPV status.^18^ Finally, these analyses regarding Medicaid coverage should be interpreted, with caution, as confirmed Medicaid payer at the time of diagnoses, given that the publicly available SEER cancer files contain missing insurance data and do not contain any insurance information on length of enrollment.^44^

## 5 CONCLUSION

This study finds little evidence that the ACA's Medicaid expansion improved early‐stage HNC detection. Rather, the improving patterns of HNC detection should be attributed to increased generosity of Medicaid dental benefits, which just happened to occur at the same time Medicaid eligibility expansion under the ACA. Only among HPV(−) HNCs in California and Washington state was there a statistically and clinically significant association between expanding Medicaid eligibility and lower rates of distant‐stage diagnoses. Until screening becomes a viable strategy, these results should also motivate approaches to decrease the burden of late‐stage HPV+ HNCs. In addition to continued examination of Medicaid eligibility and dental benefits on HNC outcomes, future policy evaluation work analyzing cancer registry data should strive to incorporate novel identification and inference procedures to motivate robust, trustworthy evidence. Whether Medicaid coverage or earlier detection lowers mortality and improves quality of life remains a critical question as the burden of HNC continues to rise.

## AUTHOR CONTRIBUTIONS

The author confirms sole responsibility for the following: study conception and design, data collection, analysis and interpretation of results, and manuscript preparation.

## FUNDING INFORMATION

National Institute of Dental and Craniofacial Research NIDCR 1F31DE032250‐01.

## CONFLICT OF INTEREST STATEMENT

The authors have stated explicitly that there are no conflicts of interest in connection with this article.

## ETHICS STATEMENT

This retrospective analysis uses deidentified, secondary data and does not meet the definition of human subjects research (45 CFR 46.101(b)(4)).

## Supporting information


**Data S1** Supporting information.

**Data S2** Supporting information.

## Data Availability

Data sharing is restricted by third‐party. Investigators can access publicly available SEER HNC‐HPV datafile at NCI.^30^
