# Supplementary material for: Examining the effect of Medicaid expansion on early detection of head and neck cancer of the oral cavity and pharynx by HPV‐type and generosity of dental benefits
Source: Cancer Rep (Hoboken). 2023 May 29;6(8):e1840. doi: 10.1002/cnr2.1840 (PMC10432424; doi:10.1002/cnr2.1840)
Supplement: Supplementary file 1 — Data S1 Supporting information. [file CNR2-6-e1840-s001.pdf]

8 Supplemental Files

Supplemental Table 1: Pre-Trend Tests, Medicaid Coverage (DD)

Treatment	HPV Status	Coefficient	Est.	[CI]		p	Joint-Test (p)
Expansion	All	2010	-0.0233	-0.1943	0.0427	0.5566	0.0601
		2011	0.0112	-0.0545	0.0500	0.7207	
		2012	-0.0171	-0.0879	0.0518	0.0821	
	HPV(-)	2010	-0.0334	-0.2616	0.0426	0.5566	0.1902
		2011	0.0007	-0.0766	0.1397	0.9930	
		2012	-0.0246	-0.0868	0.0075	0.0601	
	HPV+	2010	-0.0148	-0.1728	0.0398	0.6086	0.0561
		2011	0.0189	-0.0667	0.0415	0.6747	
		2012	-0.0136	-0.0977	0.1165	0.3083	
Expansion, Always Dental Coverage	All	2010	-0.0381	-0.1338	0.0188	0.3143	0.0501
		2011	0.0098	-0.0633	0.0507	0.7007	
		2012	-0.026	-0.0797	-0.0033	0.0400	
	HPV(-)	2010	-0.053	-0.1792	0.0534	0.2883	0.1802
		2011	0.0006	-0.1250	0.1080	0.9510	
		2012	-0.0246	-0.1059	0.0431	0.2883	
	HPV+	2010	-0.0268	-0.1150	0.0221	0.4264	0.036
		2011	0.0166	-0.0409	0.0430	0.5926	
		2012	-0.0278	-0.0879	-0.0047	0.0160	
Expansion, Added Dental Coverage in 2014	All	2010	-0.0132	-0.3312	0.0561	0.6126	0.1001
		2011	0.0114	-0.1616	0.0485	0.6827	
		2012	-0.0113	-0.1200	0.0053	0.1842	
	HPV(-)	2010	-0.0197	-0.7397	0.3603	0.5806	0.4785
		2011	-0.0020	-0.2209	0.2609	0.9409	
		2012	-0.0263	-0.1390	-0.0155	0.0220	
	HPV+	2010	-0.0071	-0.1995	0.0397	0.7508	0.2242
		2011	0.0189	-0.1437	0.0460	0.6046	
		2012	-0.0039	-0.1152	0.0203	0.8048	

Supplemental Table 1 reports the results of the pre-trend diagnostic tests for pre-2014 changes in Medicaid coverage based on the Difference-in-Differences (DD) design. Each model interacts a binary year dummy variable (2010, 2011, 2012) with state Medicaid expansion status. Using 2013 as a reference category, each estimate (Est.) tests for significant associations between the year (coefficient) and expansion status. 95% confidence intervals and respective p-values are reported based on Wild Cluster Bootstrap. The Joint-Test reports the p-value associated with the robust Wald statistic testing if all three coefficients (2010, 2011, 2012) are jointly equal to zero. Significant differences indicate a potential threat to our identification assumption of parallel trends.

Supplemental Table 2: Pre-Trend Tests, Localized Diagnosis (DD)

Treatment	HPV Status	Coefficient	Est.	[CI]		p	Joint-Test (p)
Expansion	All	2010	-0.0304	-0.1213	0.0091	0.1301	0.2182
		2011	-0.0060	-0.0514	0.0413	0.4324	
		2012	0.0057	-0.0343	-0.0243	0.5726	
	HPV(-)	2010	-0.0035	-0.1960	0.0622	0.8629	0.8529
		2011	-0.0004	-0.1703	0.0291	0.9510	
		2012	0.0132	-0.1907	0.0804	0.7387	
	HPV+	2010	-0.0489	-0.1084	0.0106	0.0701	0.0861
		2011	-0.0074	-0.1267	0.1505	0.6867	
		2012	-0.0014	-0.0421	0.1107	0.9770	
Expansion, Always Dental Coverage	All	2010	-0.0372	-0.0850	0.0054	0.0961	0.1922
		2011	-0.0069	-0.0289	0.0177	0.3744	
		2012	0.0114	-0.0067	0.0483	0.3243	
	HPV(-)	2010	-0.0243	-0.1323	0.0494	0.4164	0.3644
		2011	0.0037	-0.0978	0.0422	0.8448	
		2012	0.0050	-0.0544	0.0870	0.8689	
	HPV+	2010	-0.0502	-0.1231	0.0226	0.1201	0.1201
		2011	-0.0143	-0.0495	0.0884	0.4825	
		2012	0.0130	-0.0199	0.0959	0.4264	
Expansion, Added Dental Coverage in 2014	All	2010	-0.0260	-0.1890	0.0254	0.2162	0.4344
		2011	-0.0064	-0.1272	0.0223	0.5485	
		2012	0.0009	-0.1196	0.0392	0.9369	
	HPV(-)	2010	0.0147	-0.3166	0.0696	0.4965	0.2322
		2011	-0.0067	-0.3523	0.0204	0.7908	
		2012	0.0203	-0.3236	0.1039	0.5285	
	HPV+	2010	-0.0487	-0.1792	0.0155	0.0681	0.0280
		2011	-0.0024	-0.1151	0.1840	0.9249	
		2012	-0.0127	-0.0760	0.1140	0.4204	

Supplemental Table 2 reports the results of the pre-trend diagnostic tests for pre-2014 changes in localized-stage diagnoses based on the Difference-in-Differences (DD) design. Each model interacts a binary year dummy variable (2010, 2011, 2012) with state Medicaid expansion status. Using 2013 as a reference category, each estimate (Est.) tests for significant associations between the year (coefficient) and expansion status. 95% confidence intervals and respective p-values are reported based on Wild Cluster Bootstrap. The Joint-Test reports the p-value associated with the robust Wald statistic testing if all three coefficients (2010, 2011, 2012) are jointly equal to zero. Significant differences indicate a potential threat to our identification assumption of parallel trends.

Supplemental Table 3: Pre-Trend Tests, Distant Diagnosis (DD)

Treatment	HPV Status	Coefficient	Est.	[CI]		p	Joint-Test (p)
Expansion	All	2010	0.0195	-0.1218	-0.1134	0.5666	
		2011	0.0344	-0.0761	0.1490	0.1522	0.4665
		2012	0.0173	-0.0886	0.1683	0.5846	
	HPV(-)	2010	0.0393	-0.0566	0.2021	0.3283	
		2011	0.0413	-0.0534	0.1796	0.1001	0.9750
		2012	0.0022	-0.1084	0.3045	0.9730	
	HPV+	2010	0.0050	-0.1616	-0.1036	0.7387	
		2011	0.0261	-0.0777	0.1754	0.4705	0.1261
		2012	0.0230	-0.0907	0.0997	0.3103	
Expansion, Always Dental Coverage	All	2010	0.0127	-0.0530	0.0846	0.6426	
		2011	0.0323	-0.0256	0.1558	0.1982	0.3303
		2012	0.0059	-0.0610	0.0861	0.8248	
	HPV(-)	2010	0.0354	-0.0565	0.1832	0.4184	
		2011	0.0501	0.0007	0.1917	0.0420	0.6907
		2012	-0.0099	-0.1151	0.0957	0.8048	
	HPV+	2010	-0.0015	-0.0638	0.0408	0.9610	
		2011	0.0175	-0.0671	0.1359	0.5465	0.4104
		2012	0.0160	-0.0358	0.0854	0.4805	
Expansion, Added Dental Coverage in 2014	All	2010	0.0263	-0.0831	-0.0663	0.5305	
		2011	0.0373	-0.1121	0.1527	0.1922	0.6346
		2012	0.0265	-0.1630	0.1618	0.4645	
	HPV(-)	2010	0.0445	-0.1672	0.3352	0.3483	
		2011	0.0341	-0.2406	0.2656	0.3103	0.8068
		2012	0.0131	-0.1481	0.4279	0.7668	
	HPV+	2010	0.0116	-0.0671	0.1515	0.5866	
		2011	0.0329	-0.0949	0.1521	0.4244	0.2643
		2012	0.0291	-0.1258	0.0536	0.3263	

Supplemental Table 3 reports the results of the pre-trend diagnostic tests for pre-2014 changes in distant-stage diagnoses based on the Difference-in-Differences (DD) design. Each model interacts a binary year dummy variable (2010, 2011, 2012) with state Medicaid expansion status. Using 2013 as a reference category, each estimate (Est.) tests for significant associations between the year (coefficient) and expansion status. 95% confidence intervals and respective p-values are reported based on Wild Cluster Bootstrap. The Joint-Test reports the p-value associated with the robust Wald statistic testing if all three coefficients (2010, 2011, 2012) are jointly equal to zero. Significant differences indicate a potential threat to our identification assumption of parallel trends.

Supplemental Table 4: Pre-Trend Tests, Medicaid Coverage (DDD)

Treatment	Coefficient	Est.	[CI]		p	Joint-Test (p)
Expansion	2010	-0.0258	-0.1626	0.0320	0.1061	0.6166
	2011	-0.0165	-0.0711	0.1153	0.3864	
	2012	-0.0125	-0.0698	0.0430	0.3984	
Expansion, Always Dental Coverage	2010	-0.0336	-0.1281	0.0480	0.2342	0.5125
	2011	-0.0163	-0.1072	0.0907	0.6587	
	2012	0.0013	-0.0773	0.0759	0.9910	
Expansion, Added Dental Coverage in 2014	2010	-0.0191	-0.2318	0.0127	0.2342	0.9289
	2011	-0.0169	-0.0757	0.1986	0.4284	
	2012	-0.0225	-0.1129	0.0081	0.1602	

Supplemental Table 4 reports the results of the pre-trend diagnostic tests for pre-2014 changes in Medicaid coverage based on the Triple Differences (DDD) design. Each model interacts a binary year dummy variable (2010, 2011, 2012) with state Medicaid expansion status and an individual-level HPV(-) indicator. Using 2013 as a reference category, each estimate (Est.) tests for significant associations between the year (coefficient), expansion status, and HPV(-) status. 95% confidence intervals and respective p-values are reported based on Wild Cluster Bootstrap. The Joint-Test reports the p-value associated with the robust Wald statistic testing if all three coefficients (2010, 2011, 2012) are jointly equal to zero. Significant differences indicate a potential threat to our identification assumption of parallel trends.

Supplemental Table 5: Pre-Trend Tests, Localized Diagnosis (DDD)

Treatment	Coefficient	Est.	[CI]		p	Joint-Test (p)
Expansion	2010	0.0407	-0.1967	0.1110	0.3263	0.3083
	2011	0.0124	-0.3604	0.0915	0.7608	
	2012	0.0181	-0.2976	0.1209	0.7387	
Expansion, Always Dental Coverage	2010	0.0268	-0.1524	0.1325	0.5666	0.9429
	2011	0.0271	-0.1727	0.0973	0.5105	
	2012	-0.0025	-0.1525	0.1167	0.9690	
Expansion, Added Dental Coverage in 2014	2010	0.0526	-0.2709	0.0869	0.2563	0.0380
	2011	-0.0028	-0.5324	0.053	0.9169	
	2012	0.0334	-0.4117	0.1553	0.4805	

Supplemental Table 5 reports the results of the pre-trend diagnostic tests for pre-2014 changes in localized-stage diagnoses based on the Triple Differences (DDD) design. Each model interacts a binary year dummy variable (2010, 2011, 2012) with state Medicaid expansion status and an individual-level HPV(-) indicator. Using 2013 as a reference category, each estimate (Est.) tests for significant associations between the year (coefficient), expansion status, and HPV(-) status. 95% confidence intervals and respective p-values are reported based on Wild Cluster Bootstrap. The Joint-Test reports the p-value associated with the robust Wald statistic testing if all three coefficients (2010, 2011, 2012) are jointly equal to zero. Significant differences indicate a potential threat to our identification assumption of parallel trends.

Supplemental Table 6: Pre-Trend Tests, Distant Diagnosis (DDD)

Treatment	Coefficient	Est.	[CI]		p	Joint-Test (p)
Expansion	2010	0.0362	-0.0451	0.1919	0.0861	0.6126
	2011	0.0196	-0.1697	0.1616	0.5766	
	2012	-0.0148	-0.1163	0.2601	0.6967	
Expansion, Always Dental Coverage	2010	0.0348	-0.0598	0.1742	0.3604	0.8829
	2011	0.0412	-0.0312	0.1421	0.2603	
	2012	-0.0196	-0.1250	0.1143	0.6206	
Expansion, Added Dental Coverage in 2014	2010	0.0364	-0.0333	0.1895	0.0741	0.4725
	2011	0.0042	-0.3052	0.3135	0.9229	
	2012	-0.0104	-0.1268	0.3650	0.7508	

Supplemental Table 6 reports the results of the pre-trend diagnostic tests for pre-2014 changes in distant-stage diagnoses based on the Triple Differences (DDD) design. Each model interacts a binary year dummy variable (2010, 2011, 2012) with state Medicaid expansion status and an individual-level HPV(-) indicator. Using 2013 as a reference category, each estimate (Est.) tests for significant associations between the year (coefficient), expansion status, and HPV(-) status. 95% confidence intervals and respective p-values are reported based on Wild Cluster Bootstrap. The Joint-Test reports the p-value associated with the robust Wald statistic testing if all three coefficients (2010, 2011, 2012) are jointly equal to zero. Significant differences indicate a potential threat to our identification assumption of parallel trends.

Supplemental Table 7: Pre-Trend Tests (Adding Dental Coverage in 2014 - DD)

Treatment	HPV Status	Coefficient	Est.	[CI]		p	Joint-Test (p)
Medicaid Coverage	All	2010	0.0109	-0.0522	0.0669	0.5225	0.6867
		2011	0.0056	-0.0494	0.0525	0.7267	
		2012	0.0065	-0.0210	0.0288	0.4725	
	HPV(-)	2010	0.0150	-0.1025	0.1119	0.5786	0.6066
		2011	-0.0003	-0.1051	0.1126	0.9910	
		2012	-0.0079	-0.0548	0.0371	0.5806	
	HPV+	2010	0.0087	-0.0325	0.0481	0.4825	0.9930
		2011	0.0086	-0.0413	0.0447	0.5886	
		2012	0.0142	-0.0098	0.0328	0.2062	
Localized Dx	All	2010	-0.0018	-0.0661	0.0408	0.8649	0.8348
		2011	0.0000	-0.0572	0.0160	0.9610	
		2012	-0.0050	-0.0454	0.0162	0.5926	
	HPV(-)	2010	0.0280	-0.0474	0.0979	0.3784	0.1101
		2011	-0.0070	-0.0886	0.0236	0.6046	
		2012	0.0200	-0.0657	0.0766	0.4985	
	HPV+	2010	-0.0149	-0.0819	0.0401	0.4244	0.1061
		2011	0.0094	-0.0416	0.0418	0.6086	
		2012	-0.0197	-0.0460	0.0043	0.0661	
Distant Dx	All	2010	0.0176	-0.0341	0.0612	0.2903	0.9029
		2011	0.0157	-0.0649	0.0591	0.5285	
		2012	0.0235	-0.0446	0.0651	0.3303	
	HPV(-)	2010	0.0249	-0.0859	0.1120	0.3684	0.4044
		2011	0.0059	-0.1275	0.0583	0.7628	
		2012	0.0215	-0.0910	0.0978	0.4084	
	HPV+	2010	0.0125	-0.0237	0.0485	0.3123	0.6667
		2011	0.0199	-0.0559	0.0655	0.4384	
		2012	0.0210	-0.0449	0.0538	0.4324	

Supplemental Table 7 reports the results of the pre-trend diagnostic tests for pre-2014 changes in all oral cancer outcomes based on the Difference-in-Differences (DD) design. Each model interacts a binary year dummy variable (2010, 2011, 2012) with an indicator for whether the state added extensive dental coverage in 2014 (WA, CA). Using 2013 as a reference category, each estimate (Est.) tests for significant associations between the year (coefficient) and dental benefit change indicator. 95% confidence intervals and respective p-values are reported based on Wild Cluster Bootstrap. The Joint-Test reports the p-value associated with the robust Wald statistic testing if all three coefficients (2010, 2011, 2012) are jointly equal to zero. Significant differences indicate a potential threat to our identification assumption of parallel trends.

Supplemental Table 8: Pre-Trend Tests (Adding Dental Coverage in 2014 - DDD)

Outcome	Coefficient	Est.	[CI]		p	Joint-Test (p)
Medicaid Coverage	2010	0.0030	-0.0672	0.0729	0.8549	0.6827
	2011	-0.0083	-0.0819	0.1071	0.7367	
	2012	-0.0218	-0.0687	0.0272	0.2362	
Localized Dx	2010	0.0362	-0.0604	0.1241	0.2823	0.0300
	2011	-0.0175	-0.0959	0.0594	0.4104	
	2012	0.0358	-0.0556	0.1032	0.3443	
Distant Dx	2010	0.0137	-0.0846	0.0889	0.5526	0.2182
	2011	-0.0227	-0.1317	0.0409	0.2643	
	2012	-0.0007	-0.0812	0.0662	0.9870	

Supplemental Table 8 reports the results of the pre-trend diagnostic tests for pre-2014 changes in all oral cancer outcomes based on the Triple Differences (DDD) design. Each model interacts a binary year dummy variable (2010, 2011, 2012) with an indicator for whether the state added extensive dental coverage in 2014 (WA, CA) and an individual-level HPV(-) indicator. Using 2013 as a reference category, each estimate (Est.) tests for significant associations between the year (coefficient), dental benefit change indicator, and HPV(-) status. 95% confidence intervals and respective p-values are reported based on Wild Cluster Bootstrap. The Joint-Test reports the p-value associated with the robust Wald statistic testing if all three coefficients (2010, 2011, 2012) are jointly equal to zero. Significant differences indicate a potential threat to our identification assumption of parallel trends.
