# Supplementary material for: Examining the effect of Medicaid expansion on early detection of head and neck cancer of the oral cavity and pharynx by HPV‐type and generosity of dental benefits
Source: Cancer Rep (Hoboken). 2023 May 29;6(8):e1840. doi: 10.1002/cnr2.1840 (PMC10432424; doi:10.1002/cnr2.1840)
Supplement: Supplementary file 2 — Data S2 Supporting information. [file CNR2-6-e1840-s002.docx]

**Appendix A.**

**Supplemental Exhibit A1: Technical Appendix – Identification Assumptions and Pre-Trend Tests**

Our identifying assumption can be expressed under the potential outcomes framework as the common trends assumption:

S1) $\Delta Y\left( 1 \right)_{ist}=\Delta Y\left( 0 \right)_{ist}$

Equation S1 simply states that, in the absence of Medicaid expansion (1), the conditional change in early detection for expansion states would be similar to the change in early detection for non-expansion states (0) before and after the expansion years. The pre-treatment common trends assumption can be visually examined and empirically tested, but post-treatment common trends assumptions are unobservable counterfactuals. Here, and in all subsequent models, we test strength of the common trends assumption by specifying the following event-history design:

S2) $Y_{ist}=\beta DD_{ts}^{2010,2011,2012}+STATE_{s}+YEAR_{t}+MONTH_{m}+{X'}_{ist}+METRO_{c}+e_{ist}$

By excluding years > 2013 and using year = 2013 as a reference, equation 4 tests for differential trends in Head and Neck cancer outcomes by expansion status. We test each DD parameter for years 2010, 2011, 2012 individually and jointly (Wald).

For the Triple Difference design, no longer must we assume common trends between expansion and non-expansion states. Instead, if there are non-common trends between expansion states and non-expansion states, our new identifying assumption requires only that these non-common trends do not differ by HPV-type in ways unrelated to Medicaid expansion:

S3) $\Delta Y\left( 1 \right)_{ts}^{HPV(-)}-\Delta Y\left( 0 \right)_{ts}^{HPV(-)} =\Delta Y\left( 1 \right)_{ts}^{HPV+}\Delta Y\left( 1 \right)_{ts}^{HPV+}$

**Supplemental Exhibit A2: Pre-Trend Test Results**

Our Difference-in-Difference estimates are valid under an identification assumption that in the absence of expanding access to Medicaid dental coverage, the adjusted Head and Neck cancer trends in expansion states would be similar to trends in non-expansion states. The biggest threat to this assumption is the presence of unobserved, temporal heterogeneity in Head and Neck cancer outcomes which differs by state. The differential trend tests examine the interaction of each pre-treatment year fixed effect (2010, 2011, 2012) and treatment status (expansion). Using 2013 as the reference category, and excluding years after 2013, we test for statistically significant differences for each coefficient individually and jointly.

We observe some evidence for pre-treatment differential trends between expansion and non-expansion states. First, we find statistically significant pre-treatment coefficients and joint-tests for trends in Medicaid coverage (Supplemental Table 1). The pre-trend tests indicate that Medicaid coverage rates may have differed by expansion status in the full sample of all Head and Neck cancer patients (joint-test p = 0.0601). However, after stratifying by dental coverage generosity and HPV-type, we find that the pre-treatment differential trends in Medicaid coverage are observed for HPV+ Head and Neck cancer patients in expansion states always covering extensive dental benefits (joint-test p = 0.036).

While there also appears to be differential trends in localized-stage diagnoses, we find no evidence in differential trends in distant-stage diagnoses. Supplemental Table 2 shows pre-treatment differential trends in HPV+ localized stages for expansion states adding dental benefits at the time of expansion (joint-test p = 0.0280). For distant stage diagnoses, none of the pre-treatment joint-tests are even marginally significant at a p < 0.1 threshold (Supplemental Table 3).

The Triple-Differences design relaxes our identification assumption and now allows for differential trends by expansion status, as long as those unobserved trends are similar for HPV(-) and HPV+ Head and Neck cancers in all ways except for expanding access to Medicaid dental benefits.

Supplemental Table 4 reports the pre-treatment differential trend tests for the Medicaid coverage rates. Unlike our DD model, which estimated significant differences in pre-treatment trends, our DDD model does not include any statistically significant pre-treatment coefficients or joint-tests of differential trends in Medicaid coverage. We do, however, observe the presence of pre-treatment trends in localized stage diagnoses (Supplemental Table 5). The finding that HPV(-) trends in localized diagnoses differed from HPV+ trends in expansion states adding dental coverage in 2014 is consistent with the results of our pre-treatment trend tests for the DD model. Finally, the DDD model’s pre-treatment tests for distant-stage diagnoses do not indicate any presence of differential trends (Supplemental Table 6).

Finally, we find little evidence of pre-treatment differential trends by states adding, compared to those not changing, Medicaid dental benefits in 2014 prior to 2014. Supplemental Table 7 reports the pre-treatment differential trend tests for the DD estimates. No joint-tests were statistically significantly different than zero, providing support to the DD-design. We do observe differential trends for the DDD-estimates for localized-stage diagnoses (Supplemental Table 8).
